# The effectiveness of ultrasonography in verifying the placement of a nasogastric feeding tube in patients with low consciousness at an emergency center

**DOI:** 10.1186/2036-7902-6-S1-A16

**Published:** 2014-01-31

**Authors:** HM Kim, BH So, WJ Jeong, SM Choi, KN Park

**Affiliations:** 1 Department of Emergency Medicine, College of Medicine, The Catholic University of Korea, St. Vincent’s Hospital, Suwon, Korea; 2 Department of Emergency Medicine, College of Medicine, The Catholic University of Korea, St. Mary’s Hospital, Uijeongbu, Korea; 3 Department of Emergency Medicine, College of Medicine, The Catholic University of Korea, St. Mary’s Hospital, Seoul, Korea

## Background and objective

This study was designed to compare the effectiveness of using auscultation, pH measurements of gastric aspirates, and ultrasonography as physical examination methods to verify nasogastric tube placement in emergency room patients with low consciousness who require nasogastric tube insertion.

## Patients and methods

The study included 47 patients who were all over 18 years of age. In all patients, tube placement was verified by chest x-rays. Auscultation, pH analysis of gastric aspirates, and ultrasonography were conducted on each patient in random order. The mean patient age was 57.62 ± 17.24 years, and 28 males (59.6%) and 19 females (40.4%) were included. The nasogastric tube was inserted by an emergency room resident. For pH testing, gastric aspirates were dropped onto litmus paper, and the resulting color of the paper was compared with a reference table. Ultrasonography was performed by an emergency medicine specialist, and the chest x-ray examination was interpreted by a different emergency medicine specialist who did not conduct the ultrasonography test. The results of the auscultation, gastric aspirate pH, and ultrasonography examinations were compared with the results of the chest x-ray examination.

## Results

The sensitivity and specificity were 100% and 33.3%, respectively, for auscultation and 86.4% and 66.7%, respectively, for ultrasonography. Kappa values were the highest for auscultation at 0.484 compared to chest x-rays, followed by 0.299 for ultrasonography and 0.130 for pH analysis of the gastric aspirate. However, the p value for the pH method was 0.182 (>0.05).

## Conclusion

Ultrasonography is useful for confirming the results of auscultation after nasogastric tube insertion among patients with low consciousness at an emergency center. When ultrasound findings suggest that the nasogastric tube placement is not gastric, additional radiographic examinations should be performed.

